# CBX4 Provides an Alternate Mode of Colon Cancer Development *via* Potential Influences on Circadian Rhythm and Immune Infiltration

**DOI:** 10.3389/fcell.2021.669254

**Published:** 2021-06-07

**Authors:** Wangzhi Wei, Wei Zhao, Yu Zhang

**Affiliations:** Life Science Institute of Jinzhou Medical University, Jinzhou, China

**Keywords:** chromobox 4 (CBX4), circadian rhythm, circadian clock gene, colon cancer, immune infiltration

## Abstract

The circadian machinery is critical for the normal physiological functions and cellular processes. Circadian rhythm disruption has been associated with immune suppression which leads to higher cancer risk, suggesting a putative tumor protective role of circadian clock homeostasis. CBX4, as an epigenetic regulator, has been explored for its involvement in tumorigenesis. However, little is known about the correlation between CBX4 and circadian rhythm disruption in colon cancer as well as the potential impact on the tumor immunity. A significant upregulation of CBX4 was identified in the TCGA colon adenocarcinoma (COAD) samples when compared with the normal controls (*p* < 0.001). This differential expression was confirmed at the protein level using colon adenocarcinoma tissue array (*p* < 0.01). CBX4 was up-regulated in the recurred/progressed colon cancer cases compared with the disease-free samples *(p* < 0.01), suggesting CBX4 as a potential predictor for poor prognosis. With regard to nodular metastasis, CBX4 was found to be associated with early onset of metastatic diseases but not late progression. The circadian rhythm is orchestrated by the alternating activation and suppression of the CLOCK/ARNTL-driven positive loop and the PER/CRY-controlled negative loop. In COAD, CBX4 was negatively correlated with CLOCK (*p* < 0.001), and positively correlated with PER1 (*p* < 0.001), PER3 (*p* < 0.01), and CRY2 (*p* < 0.001) as well as NR1D1 (*p* < 0.001), a critical negative regulator of the circadian clock. These interactions consistently impacted on patient survival based on the colorectal cancer cohorts GSE17536 and GSE14333 of PrognoScan. CBX4 showed significant negative correlations with infiltrating B cells (*p* < 0.05) and CD4^+^ T cells (*p* < 0.01), and positive correlations with myeloid derived suppressor cells (MDSCs) (*p* < 0.05) and cancer associated fibroblast (CAFs) (*p* < 0.001), as well as a low immunoscore. Moreover, CBX4 displayed significant correlations with diverse immune metagenes. PER1 and PER3, consistent with their coordinated expression with CBX4, also had strong correlations with these gene representatives in COAD, suggesting a potential interaction of CBX4 with the circadian machinery. Our studies implicate that CBX4 may contribute to colon cancer development via potential influence on circadian rhythm and immune infiltration. These findings provide new insights into deciphering the function of CBX4, and may contribute to the development of new targeting strategies.

## Introduction

Colorectal carcinoma (CRC) is one of the leading causes of morbidity and mortality worldwide. It accounted for 9.2% of total cancer deaths in 2018 (Bray et al., 2018). For incidence, it is the second frequent burden of cancer among females, and the third of that among males (Bray et al., 2018). Screening has been demonstrated to reduce both incidence and mortality of CRC (Zauber, 2015), and targeting of essential oncogenes represents an effective way for the therapies. Although numerous potential targets have been identified in CRC, the biological and molecular mechanisms underlying CRC development and progression are still far from being fully understood (Malki et al., 2020).

Polycomb group (PcG) proteins act as important transcriptional repressors that mediate epigenetic gene silencing through histone modification (Simon and Kingston, 2009). Two distinct polycomb repressive complexes (PRC), PRC1 and PRC2, play a critical role in regulation of cellular functions (Sauvageau and Sauvageau, 2010). Several polycomb chromobox (CBX) proteins including CBX2, CBX4, CBX6, CBX7, and CBX8, interact with the core PRC1 complex (Vandamme et al., 2011). The chromodomain in the N terminus and two SUMO-interacting motifs (SIMs) of CBX4 protein contribute to its polycomb- and SUMO E3 ligase-dependent functions, respectively (Luis et al., 2011). CBX4 plays oncogenic or tumor suppressive effects in a cell type dependent manner (Li et al., 2014; Wang et al., 2016; Meng et al., 2018). Despite extensive characterization of the potential mechanisms of CBX4-mediated tumorigenesis, additional partners engaged by CBX4 remain to be explored. When we focused on in-depth exploration of CBX4’s functional panorama, an interesting potential connection between CBX4 and circadian rhythm attracted our attention.

The 2017 Nobel Prize in Physiology or Medicine was awarded to Jeffrey C. Hall, Michael Rosbash and Michael W. Young for their leading discoveries of the molecular mechanisms controlling the circadian rhythm (Burki, 2017). The circadian machinery governs a remarkable variety of physiological functions and cellular processes. It is orchestrated by the regulatory feedback loops of core clock genes, which are comprised of bHLH-PAS transcription factors CLOCK (Circadian Locomoter Output Cycles Kaput) and ARNTL (Aryl Hydrocarbon Receptor Nuclear Translocator Like), as well as their downstream clock-controlled targets including Period (*Per1, Per2, Per3*) and Cryptochrome (*Cry1 and Cry2*). In the morning, the CLOCK:ARNTL heterodimer activates the transcription of PER, CRY and other Clock controlled genes (CCGs). Late in the day, PER and CRY proteins accumulate and translocate from the cytoplasm to the nucleus, where they associate and negatively regulate the circadian machinery by inhibiting the CLOCK:ARNTL complex. The alternating activation and suppression of the CLOCK:ARNT -driven positive loop and the PER/CRY-controlled negative loop result in a circadian oscillation of the molecular clock (Kettner et al., 2016; Papagiannakopoulos et al., 2016). Several studies in line with the epidemiological studies have documented the association of dysregulated circadian rhythms and increased susceptibility for developing multiple malignancies including breast (Lesicka et al., 2018; Lin and Farkas, 2018), colon (Innominato et al., 2009; Fuhr et al., 2019), prostate (Flynn-Evans et al., 2013; Wendeu-Foyet and Menegaux, 2017), lung (Papagiannakopoulos et al., 2016) cancers and hepatocellular carcinoma (Kettner et al., 2016). For example, the expression of the *Per* genes was deregulated in breast cancer and non-small lung cancer cells (Chen et al., 2005; Gery et al., 2007). Mice lacking the circadian genes were reported to be cancer prone (Lee et al., 2010). In addition, the rhythmic control of cell fate was found to affect cancer therapies (Dallmann et al., 2016; Lesicka et al., 2018).

The immune system imposes a profound impact on tumor progression through infiltration of both innate and adaptive immune cells into tumor microenvironment. Interactions between tumor cells and neighboring infiltrating cells affect cancer cell phenotype as well as neoplastic outcome (Colangelo et al., 2017). Immune functions are subject to the regulation of circadian rhythm. For example, leukocyte trafficking occurs in a circadian manner, and the cyclical recruitment of immune cells to tissues is critical for immune responses (Scheiermann et al., 2013). Perturbation of circadian clock can cause immune suppression and increased cancer risk, supporting a putative tumor protective role of circadian clock homeostasis (Savvidis and Koutsilieris, 2012; Labrecque and Cermakian, 2015).

Despite still a preliminary stage of this study, we anticipate to provide a new functional implication of CBX4 and direct more researchers toward the delineation of the mechanisms linking circadian rhythm disruption to cancers, which is a major public health issue that has yet to receive the recognition it deserves.

## Materials and Methods

### Tumor Immune Estimation Resource (TIMER) Analyses

Tumor Immune Estimation Resource (TIMER) is a comprehensive resource for analysis of molecular characterization of tumor-immune interactions (Li et al., 2016, 2017). Human CBX4 expression was screened via the DiffExp module of TIMER. Distributions of the CBX4 expression levels were displayed in the box plots and the statistical significance was evaluated by Wilcoxon test. Up- or down- regulation of CBX4 in the tumors was compared to normal tissues for each cancer type.

Correlations between CBX4 expression and abundance of the immune infiltrates were investigated via the Gene module of TIMER2.0, which integrates multiple algorithms for immune infiltration estimation instead of only using one algorithm in the original TIMER (Li et al., 2020). The partial Spearman’s correlation was performed by selecting the “Purity Adjustment” option. Genes highly expressed in tumor microenvironment intend to have negative associations with tumor purity, while those with positive associations are expected to be highly expressed in tumor cells (Aran et al., 2015; Li et al., 2017).

Correlations between CBX4 and the related markers were explored via the gene correlation module of TIMER2. The scatter plots were generated using CBX4 expression as the Y-axis and the related makers as the X-axis, together with the partial Spearman’s rho coefficient and the statistical significance. The gene expression levels were displayed with log2 TPM.

Influences of the immune infiltrating subsets on the clinical outcome of colon adenocarcinoma (COAD) were evaluated by the Survival module. The Kaplan-Meier plots were output for B cells, CD4 + Th2 cells, MDSC, and CAFs to visualize the survival differences between the high and low infiltration levels. The hazard ratio and p value for Cox model and the log-rank p value for Kaplan-Meier curve were shown.

### Queries of TCGA Data *via* Cbioportal and UALCAN Web Portals

Influences of CBX4 on clinicopathologic features of colon cancer were evaluated by UALCAN^1^, an interactive web tool not only to compare primary tumor with normal tissue samples, but also to analyze different tumor subgroups as defined by individual cancer stages, histological subtypes, nodular metastasis status, and other clinical parameters based on TCGA level 3 RNA-seq and clinical data from 31 cancer types (Chandrashekar et al., 2017). The individual cancer stages are categorized into Stage I (T1 or T2 N0 M0); Stage II (T3 or T4 N0 M0); Stage III (Tx N1 or N2 M0); Stage IV(Tx Nx M1) (Chandrashekar et al., 2017). The histological subtypes are divided into adenocarcinoma and mucus adenocarcinoma, and the nodular metastasis status is grouped into N0 (No regional lymph node metastasis); N1 (Metastases in 1 to 3 axillary lymph nodes); N2 (Metastases in 4 to 9 axillary lymph nodes) (Chandrashekar et al., 2017).

The cBioPortal for Cancer Genomics^2^ provides a web resource for integrative analysis of cancer genomics and clinical profiles (Cerami et al., 2012; Gao et al., 2013). A histogram of alteration frequencies of CBX4 across cancers, including mutations, amplifications and deletions, was summarized based on TCGA Pan Cancer Atlas studies in which 594 of the colorectal carcinoma cases were included. A frequently mutated gene (FMG) is defined as a mutation frequency greater than 5% in a type of cancer. Using the “*Co-expression*,” the correlations between CBX4 expression (log RNA Seq V2 RSEM) and the circadian clock genes (log RNA Seq V2 RSEM) were displayed in the scatter plots. Spearman’s and Pearson’s coefficients were indicated with significance. Using the “*Plot*,” levels of CBX4 mRNA expression (z scores relative to normal samples) (*n* = 222) were compared between the recurred/progressed colon cancer cases (*n* = 29) and the disease-free samples (*n* = 193). The data were collected from the clinical attribute of the study.

### Tissue Array Immunohistochemistry

Formalin-fixed paraffin-embedded tissue array sections including a total of 101 cases of primary colon adenocarcinoma (Catalog# HColA180Su17) were obtained from Outdo Biotech (Shanghai, China). The sections were dewaxed in five changes of xylene, rehydrated through graded alcohols, and heat treated in Tris-EDTA buffer at pH 9.0 for antigen retrieval. Endogenous peroxidase activity was blocked using 3% hydrogen peroxide. After blocking with 2.5% normal serum for 20 min, the sections were then incubated with 1:200 diluted CBX4 antibody (Abcam) for 1hr at RT, followed by incubation with ImmPRESS horse anti-rabbit IgG polymer reagent for 30 min as per kit instructions (Vector Laboratory). After wash in Tris buffered saline for 2 × 5 min, the reaction was visualized with DAB (DAKO). The sections were counterstained with Mayer’s hematoxylin, dehydrated in alcohols, cleared in xylene, and mounted with MM24 Leica mounting medium (VWR).

Each patient was represented by one core of adenocarcinoma sample (*n* = 93), and the majority contained one core of non-neoplastic colon tissue control (*n* = 71). The evaluation of CBX4 expression levels was based on a staining intensity score (i) and a distribution score (Pi). The staining intensity was classified into four levels, namely 0, 1+, 2+, and 3+, which were designated as negative, weakly positive, moderately positive, and strongly positive signals, respectively; The percentage of stained cells was categorized as 0 (0%), 1(< 25%), 2 (25–50%), 3 (50–75%), and 4 (>75%), respectively. The total score Pi^∗^ (i + 1) was calculated by multiplying the intensity score by the corresponding distribution score. A total score of eight was used as a cutoff to compare the dichotomized cases with high and low expression of CBX4.

### STRING Database

STRING database provides access to predict functional protein-protein interactions (PPI) (von Mering et al., 2003; Szklarczyk et al., 2019). The associated PPI network of CBX4 pertaining to Homo sapiens was constructed by STRING v11.0 based on a highest confidence coefficient defined by a minimum interaction score >0.9. All the active interaction parameters were selected, including text-mining, experiments, databases, co-expression, neighborhood, gene fusion, and co-occurrence. 16 of STRING interactants for CBX4 was summarized with PPI enrichment p-value: <1.0 × 10^–16^.

### PrognoScan Survival Analysis

PrognoScan evaluates prognostic value of potential tumor makers or therapeutic targets using minimum P-value approach to find optimal cutpoints (Mizuno et al., 2009). Relationships between gene expression and patient endpoints, such as overall survival (OS) and disease-free survival (DFS) are analyzed according to a collection of public cancer microarray datasets with clinical annotation (Mizuno et al., 2009). For each optimal cutpoint, patients are dichotomized and survival difference between high and low expression groups is calculated by log-rank test. Here, Kaplan-Meier survival curves comparing the high and low expression of CBX4 as well as the circadian rhythm genes in colorectal carcinoma, were generated based on two colorectal cancer cohorts GSE17536 (Probe ID: 227558_at CBX4; 217563 at CLOCK; 202861 at PER1; 212695_at CRY2) and GSE14333 (Probe ID: 206724_at CBX4, 1569701_at PER3). The threshold was adjusted to a Cox P-value. 95% confidence intervals (CI) for each group were indicated by dotted lines in each plot.

### Gene Correlation Analysis in GEPIA

GEPIA (Gene Expression Profiling Interactive Analysis) includes 9,736 tumors and 8,587 normal samples from TCGA and the GTEx projects. It can be divided into several major functions including pairwise gene correlation analysis (Tang et al., 2017). Correlations between CBX4 and the related markers were explored, and the Spearman’s and Pearson’s correlation coefficients were indicated. The COAD tumor and normal colon tissue data sets were used for the analysis.

### Estimation of Infiltrating Immune Cells by ESTIMATE Algorithm

ESTIMATE (Estimation of STromal and Immune cells in MAlignant Tumor tissues using Expression data) is used as an algorithm to calculate stromal and immune scores to infer the levels of infiltrating stromal and immune cells which are major non-tumor constituents of tumor samples (Yoshihara et al., 2013). 210 of TCGA samples were divided into two groups based on CBX4 mRNA expression levels (FPKM). The corresponding immune scores were profiled from the RNA-seq-V2 platform of the ESTIMATE database and analyzed based on the dichotomized cases with high and low expression of CBX4.

### Statistical Analyses

**FIGURE 1:**
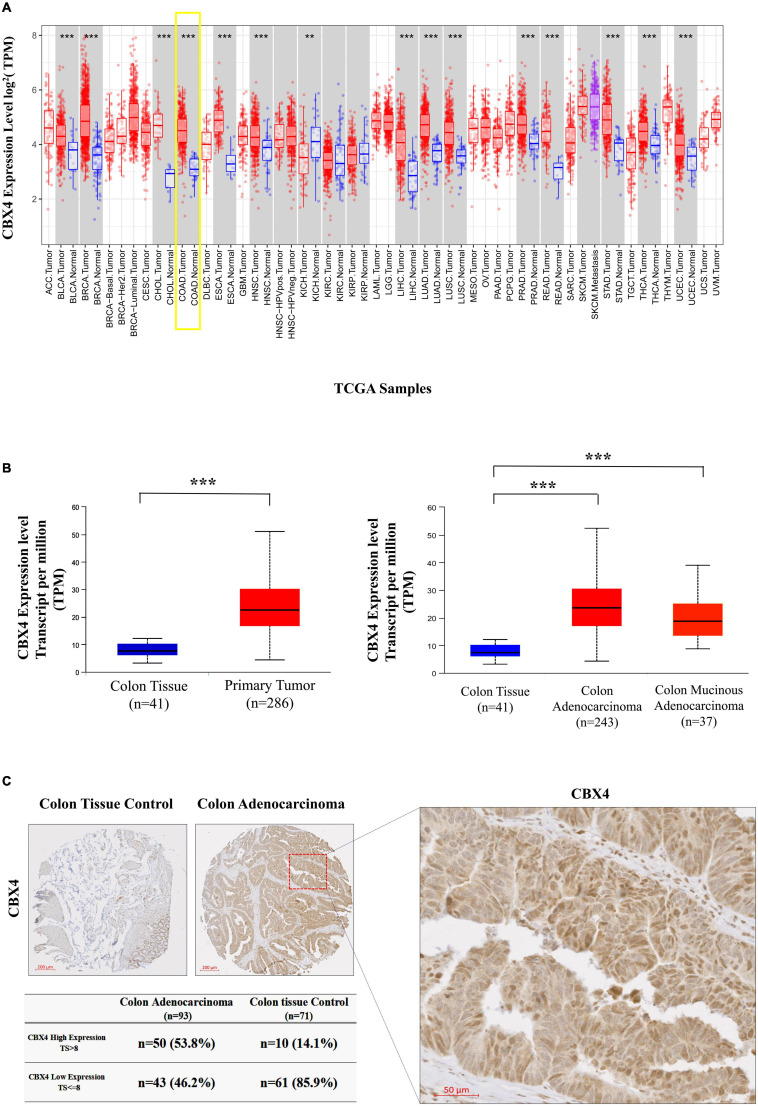
CBX4 is Preferentially Expressed in Human Colon Adenocarcinoma (COAD). **(A)** Human CBX4 expression levels in different tumor types derived from TCGA were determined by the Diff Exp module of TIMER. Red bars represent tumor tissues and blue bars are adjacent normal tissues. The gray columns here mean “normal control data are available”. The significant up- or down- regulation of CBX4 in the tumors, when compared to normal tissues, was indicated with the symbols (*P < 0.05, **P < 0.01, and ***P < 0.001). **(B)** CBX4 expression levels in COAD were analyzed by UALCAN, box plots (left) showing the levels of CBX4 in normal (*n* = 41) and colon cancer samples (*n* = 286), and box plots (right) showing those of CBX4 in normal (*n* = 41), colon adenocarcinoma (*n* = 243) and colon mucinous adenocarcinoma (*n* = 37). The read counts were normalized in TPM (Transcripts Per Kilobase Million). **(C)** Immunohistochemitry on a total of 93 cases of primary colon adenocarcinoma. Each patient was represented by one core of adenocarcinoma sample (*n* = 93), and the majority contained one core of non-neoplastic colon tissue control (*n* = 71). By taking into consideration of a intensity score (i) and a distribution score (Pi), a total score (TS) = Pi* (i + 1) was assigned for each sample using a score of 8 as a cutoff to compare the dichotomized cases with high and low expression of CBX4.

**FIGURE 2:**
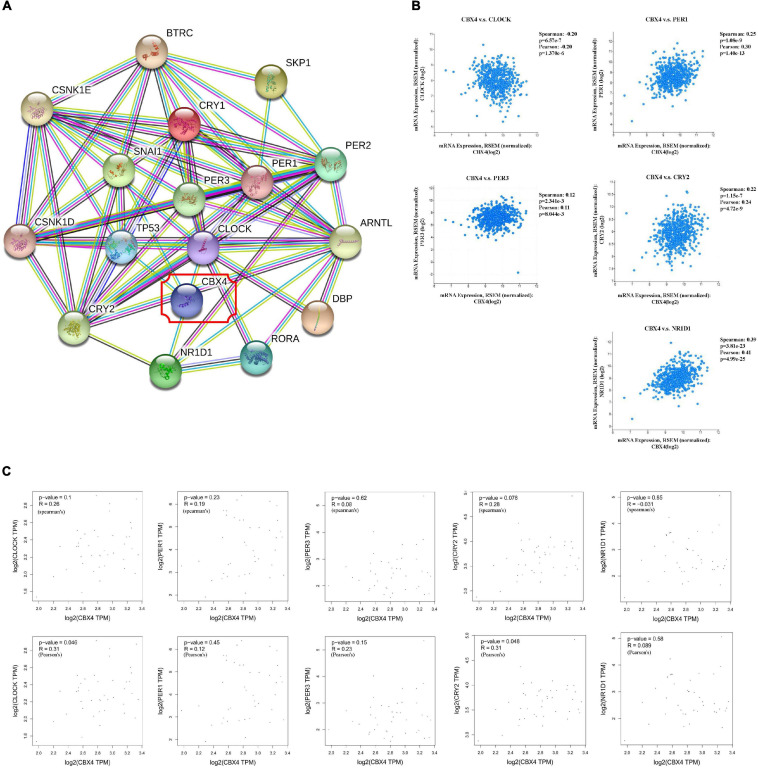
CBX4 Expression Correlates with That of Circadian Clock Genes in Colon Adenocarcinoma. **(A)** The associated PPI network of CBX4 pertaining to Homo sapiens was constructed by STRING v11.0 based on a highest confidence coefficient defined by a minimum interaction score >0.9. 16 of STRING interactants for CBX4 were summarized, PPI enrichment p-value: <1.0 × 10^–16^. **(B)** The correlations between CBX4 and the circadian clock genes including CLOCK, PER1, PER3, CRY2, NR1D1 in COAD samples were determined by cBioportal. Levels of the mRNA expression were normalized in Log2 RSEM. The correlation coefficients and the significance were indicated. **(C)** The correlations between CBX4 and the circadian genes in normal colon tissue samples were examined by GEPIA database. The gene expression levels were shown in TPM. Spearman’s (upper) and Pearson’s (lower) R and the significance were indicated.

**FIGURE 3:**
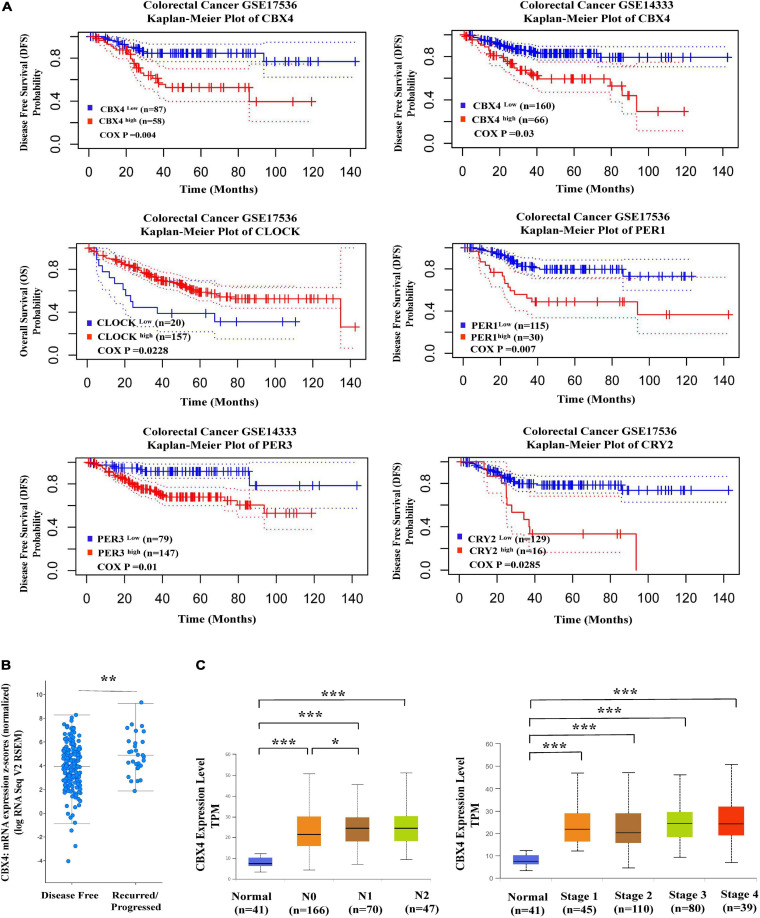
Elevated CBX4 expression predicts poor prognosis in Colon Carcinoma. **(A)** Shown (upper) are the Kaplan-Meier survival curves comparing the high and low expression of CBX4 in colorectal carcinoma generated by the PrognoScan database. Disease Free Survival (DFS) in two colorectal cancer cohorts GSE17536 (*n* = 145) and GSE14333 (*n* = 226) was analyzed. Shown (lower) are the Kaplan-Meier survival curves comparing the high and low expression of the core clock gene PER1, PER3, CLOCK, and CRY2. DFS or Overall survival (OS) in two colorectal cancer cohorts GSE17536 and GSE14333 was analyzed. The X-axis represents months to follow-up and the Y axis is survival rate, 95% confidence intervals for each group are indicated by dotted lines. **(B)** Based on the study of Colorectal Adenocarcinoma (TCGA, PanCancer Atlas), levels of CBX4 mRNA expression (log RNA Seq V2 RSEM) (*n* = 222) were compared between the recurred/progressed colon cancer cases (*n* = 29) and the disease-free samples (*n* = 193). The data were collected from the clinical attribute of the study. The vertical axis represents CBX4 mRNA expression (z scores relative to normal samples), and the horizontal axis is the categories of disease free status. **(C)** The correlation of CBX4 expression with colon adenocarcinma metastasis was analyzed using UALCAN. Levels of CBX4 expression was normalized in TPM. For the nodular metastasis status, N0 is no regional lymph node metastasis; N1 is Metastases in 1 to 3 axillary lymph nodes; N2 is Metastases in 4 to 9 axillary lymph nodes. For the cancer stages, Stage I: T1 or T2 N0 M0; Stage II – T3 or T4 N0 M0; Stage III – Tx N1 or N2 M0; Stage IV – Tx Nx M1. * *P* < 0.05, ** *P* < 0.01, *** *P* < 0.001.

**FIGURE 4:**
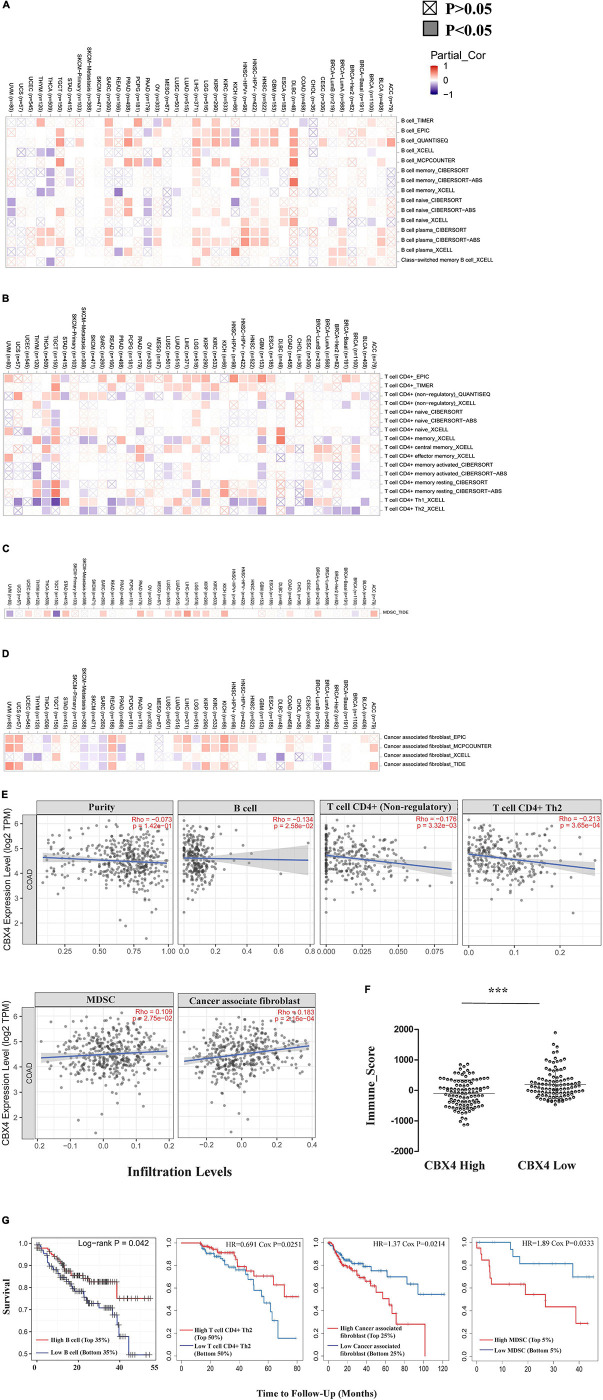
CBX4 Impacts Immunophenotype in Colon Cancer. Correlations between CBX4 expression and the infiltration abundance of B cells **(A)**, CD4 + T cells **(B)**, myeloid derived suppressor cells (MDSCs) **(C)** and cancer associated fibroblast (CAFs) **(D)** were assessed in the 40 cancer types from the Gene module of TIMER2.0. The heat maps integrated multiple algorithms for immune infiltration estimation and showed the purity-adjusted partial spearman’s rho across various cancer types. Positive correlations (rho > 0) are red and negative correlations (rho < 0) are blue. The significance of these correlations was classified into *P* < 0.05, shown in solid squares and *P* > 0.05, shown in hollow squares. **(E)** In COAD (*n* = 458), scatter plots showed the relationship between CBX4 expression in log2 TPM and levels of tumor purity, infiltrating B cells, CD4 + T cells, MDSCs, and CAFs. Spearman’s rho and statistical significance were shown. **(F)** The immune score of ESTIMATE algorithm representing the overall infiltration levels of the immune cells in COAD. 210 of TCGA samples were divided into two groups based on the FPKM levels of CBX4. The immune scores for these samples were collected from the RNAseq V2 platform of the ESTIMATE database, and analyzed based on the dichotomized cases with high and low expression of CBX4. * *P* < 0.05, ** *P* < 0.01, *** *P* < 0.001 **(G)** Influences of the above immune infiltrating subsets on the clinical outcome of COAD were evaluated by the Survival module of TIMER. The Kaplan-Meier plots visualized the survival differences between the high and low infiltration levels of B cells, CD4 + Th2 cells, MDSCs and CAFs. The hazard ratio and p value for Cox model and the log-rank p value for Kaplan-Meier curve were shown.

Distributions of the CBX4 expression levels were evaluated by the Wilcoxon test (Figure 1A); The comparisons were determined by *T*-test in Figures 1B, 3B,C, 4F; Chi-square and Fisher’s exact test were used for the tissue array analysis in Figure 1C; Spearman’s and/or Pearson’s correlation analysis were performed in Figures 2, 4, Tables 1–3 as well as Supplementary Figures 2, 3; Log-rank test and Kaplan-Meier estimators were implemented for survival analysis in Figures 3A, 4G. Significance was shown as *P < 0.05, **P < 0.01, ***P < 0.001.

**TABLE 1:** Correlations between CBX4 and gene representatives of various functional cell subsets in COAD determined by TIMER2.0.

	Colon adenocarcinoma (COAD)		
	
Cell type	Gene marker	CBX4 Correlation (TIMER 2.0)	

		Purity-adjusted partial	*P* Value
		spearman’s rho value	
T cell exhaustion	PD-1(PDCD1)	0.152	**
	CTLA4	0.093	0.062
	LAG3	0.138	**
	TIM-3 (HAVCR2)	0.066	0.182
Regulatory T cells	FOXP3	0.127	*
	TGFβ (TGFB1)	0.225	***
	L1CAM	0.469	***
	PTGIR	0.238	***
	ITGA4	0.016	0.743
Th1 cells	T-bet (TBX21)	0.119	*
	STAT1	0.197	***
	STAT4	0.02	0.687
	IFN-γ (IFNG)	0.076	0.126
Th2 cells	GATA3	0.197	***
	STAT6	0.253	***
Th17 cells	IL-6R	0.203	***
	STAT3	0.221	***
	RORγt (RORC)	-0.035	0.479
	IL-17A	-0.071	0.154
Macrophage	CD68	0.174	***
MDSC	CD11b (ITGAM)	0.139	**
	IL-4R	0.286	***
	CD33	−0.006	0.902
	CD14	0.126	*
	S100A9	−0.007	0.881

*P < 0.05, **P < 0.01, and ***P < 0.001.

**TABLE 2:** Correlations between CBX4 and gene representatives of various functional cell subsets in COAD determined by GEPIA.

Immune markers	CBX4 Correlation (GEPIA)							
	
	COAD Tumor				COAD Normal			
		
	Spearman’s	*p* value	Pearson’s	*p* value	Spearman’s	*p* value	Pearson’s	*p* value
PD-1(PDCD1)	0.17	**	0.17	**	0.28	0.079	0.38	0.015
LAG3	0.15	*	0.21	***	0.15	0.34	0.19	0.24
FOXP3	0.12	*	0.12	*	0.2	0.21	0.27	0.089
TGFβ (TGFB1)	0.27	***	0.33	***	0.65	***	0.71	***
L1CAM	0.44	***	0.32	***	0.15	0.37	0.24	0.14
PTGIR	0.25	***	0.14	*	0.16	0.32	0.25	0.11
T-bet (TBX21)	0.15	*	0.15	*	0.16	0.32	0.2	0.2
STAT1	0.24	***	0.3	***	0.096	0.55	−0.09	0.58
GATA3	0.19	**	0.15	*	0.36	*	0.41	**
STAT6	0.23	***	0.19	**	0.42	**	0.44	**
IL-6R	0.22	***	0.13	*	−0.22	0.18	−0.16	0.33
STAT3	0.27	***	0.25	***	0.37	*	0.25	0.11
CD68	0.18	**	0.22	***	0.097	0.55	0.068	0.67
CD11b (ITGAM)	0.2	***	0.13	*	0.51	***	0.4	**
IL-4R	0.27	***	0.21	***	0.2	0.2	0.29	0.064

*P < 0.05, **P < 0.01, and ***P < 0.001.

**TABLE 3:** Correlations between PER1, PER3, and gene representatives of various functional cell subsets in COAD determined by TIMER2.0.

	Colon Adenocarcinoma (COAD)				
	
Cell type	Gene marker	PER1		PER3	
			
		spearman’s rho	*P* Value	spearman’s rho	*P* Value
T cell exhaustion	PD-1(PDCD1)	0.272	***	0.246	***
	CTLA4	0.305	***	0.259	***
	LAG3	0.309	***	0.257	***
	TIM-3 (HAVCR2)	0.258	***	0.255	***
Regulatory T cells	FOXP3	0.269	***	0.286	***
	TGFβ (TGFB1)	0.34	***	0.231	***
	L1CAM	0.189	***	0.147	**
	PTGIR	0.315	***	0.214	***
	ITGA4	0.205	***	0.342	***
Th1 cells	T-bet (TBX21)	0.303	***	0.287	***
	STAT1	0.308	***	0.351	***
	STAT4	0.224	***	0.305	***
	IFN-γ (IFNG)	0.122	*	0.209	***
Th2 cells	GATA3	0.245	***	0.282	***
	STAT6	0.195	***	0.229	***
Th17 cells	IL-6R	0.209	***	0.395	***
	STAT3	0.282	***	0.406	***
	RORγt (RORC)	−0.087	0.08	0.145	**
	IL-17A	−0.231	***	−0.072	0.15
Macrophage	CD68	0.254	***	0.277	***
MDSC	CD11b (ITGAM)	0.232	***	0.244	***
	IL-4R	0.413	***	0.354	***
	CD33	0.146	**	0.223	***
	CD14	0.239	***	0.228	***
	S100A9	0.01	0.834	−0.031	0.539

*P < 0.05, **P < 0.01, and ***P < 0.001.

## Results

### CBX4 Is Preferentially Expressed in Human Colon Adenocarcinoma

Elusive documentations and paradoxical effects of CBX4 in colon cancer provoked our further investigations on its actions. Since CBX4 plays a critical role for physiological functioning, we thus analyzed the potential molecular alterations of this gene during colorectal carcinogenesis. As shown in Supplementary Figure 1, CBX4 gene alteration frequencies varied notably among cancers. In colorectal carcinoma, the mutation frequency was lower than 5%, a threshold usually used to define a FMG. As much as the copy number variation was concerned, amplification or deletion was rarely observed in colorectal carcinoma. Hence, CBX4 appears to be relatively stable at the genomic level.

The low frequencies of the genomic alterations in CBX4 indicated that the abnormalities might be regulated at the expression level. Therefore, we performed a pan-cancer screening of CBX4 expression via the DiffExp module of TIMER which allows to compare differential expression of a gene of interest between tumors and adjacent normal tissues across the TCGA malignancies. A significant up-regulation of CBX4 was identified in COAD when compared with normal controls (*p* < 0.001) (Figure 1A). UALCAN analysis confirmed the up-regulation of CBX4 in primary COAD tumor (*n* = 286) versus normal tissue samples (*n* = 41) (*p* < 0.001). Of note, the increased expression was seen in both colon adenocarcinoma (*n* = 243) and colon mucinous adenocarcinoma (*n* = 37) (*p* < 0.001) (Figure 1B).

Next, we explored CBX4 protein expression using a tissue array composed of 71 normal colon tissues and 93 cancer samples. CBX4 was found to be preferentially expressed in glandular cells with intracellular localization (Figure 1C). By taking into consideration of staining intensity and the percentage of stained cells, a score was assigned for each sample. Using a score of 8 as cutoff, 53.8% of the adenocarcinoma samples demonstrated a high level of CBX4 protein, whereas only 14.1% of the non-neoplastic colon tissues showed high expression (*p* < 0.01). Taken together, these data support a significant up-regulation of CBX4 gene expression in colon adenocarcinoma.

### CBX4 Expression Correlates With That of Circadian Clock Genes in Colon Adenocarcinoma

To understand the underlying molecular mechanisms and actions of CBX4 in colon cancer, functional protein-protein interaction (PPI) predictions by STRING were performed. The associated PPI network of CBX4 pertaining to Homo sapiens was constructed based on a highest confidence coefficient defined by a minimum interaction score >0.9. Notably, several components of the network were associated with circadian clock genes (Figure 2A). To further explore the connection between CBX4 and the circadian machinery, 594 of colorectal adenocarcinoma samples from TCGA were accessed to perform a query for CBX4 via cBioportal. Coordinated expression of CBX4 was revealed with several circadian clock genes (Figure 2B). CLOCK, one of the major activators that drive the positive regulatory loop of circadian rhythm, was negatively correlated with CBX4 (*p* < 0.001). On the contrary, PER1 (*p* < 0.001), PER3 (*p* < 0.01), and CRY2 (*p* < 0.001), members of the PER/CRY-controlled negative loop of the circadian clock, were positively correlated with CBX4. A positive correlation was also identified with NR1D1 (*p* < 0.001), a critical negative regulator of circadian rhythm directly repressing the core clock components (Polidarova et al., 2017). These results suggested such a possibility that up-regulated CBX4 expression in COAD might be cooperated with altered circadian clock gene actions. Furthermore, the relationship between CBX4 and the circadian regulators was tested in normal colon tissues via GEPIA. None of the significant correlations described above was observed in normal tissues (Figure 2C), suggesting that these interactive events may be specifically involved, and represent functional significance in tumor environment.

### Elevated CBX4 Expression Predicts Poor Prognosis in Colon Carcinoma

To reveal the prognostic potential of CBX4 in colon cancer, DFS in two colorectal cancer cohorts GSE17536 (*n* = 145) and GSE14333 (*n* = 226) was analyzed, comparing the dichotomized cases with high and low expression of CBX4. In both cohorts, increased expression of CBX4 was associated with significant poor DFS (Figure 3A upper). In line with these findings, cBioportal analysis demonstrated that cancer samples from the recurred/progressed patients had higher CBX4 mRNA levels than those from the disease-free patients (Figure 3B). Furthermore, we interrogated the correlation of CBX4 expression with cancer metastasis using UALCAN. The nodular metastasis status was grouped into N0 (*n* = 166), N1 (*n* = 70) and N2 (*n* = 47). CBX4 expression levels were compared among differential groups and the statistical significance was shown as follows: *P*_Normal–vs–N__0_ < 0.001; *P*_Normal–vs–N__1_ < 0.001; *P*_Normal–vs–N__2_ < 0.001; *P*_N__0__–vs–N__1_ < 0.05; *P*_N__0__–vs–N__2_ = 0.12; *P*_N__1__–vs–N__2_ = 0.876 (Figure 3C left). It is interesting to note that altered CBX4 expression primarily occurred during the early onset instead of the progressive stages. Similarly, CBX4 expression levels were evaluated against cancer stages which are categorized into normal (*n* = 41), stage I (*n* = 45), stage II (*n* = 110), stage III (*n* = 80), and stage IV (*n* = 39), and the statistical significance was shown as follows: *P*_Normal–vs–Stage__1_ < 0.001; *P*_Normal–vs–Stage__2_ < 0.001; *P*_Normal–vs–Stage__3_ < 0.001; *P*_Normal–vs–Stage__4_ < 0.001; *P*_Stage__1__–vs–Stage__2_ = 0.73; *P*_Stage__1__–vs–Stage__3_ = 0.053; *P*_Stage__1__–vs–Stage__4_ = 0.087; *P*_Stage__2__–vs–Stage__3_ = 0.0786; *P*_Stage__2__–vs–Stage__4_ = 0.1; *P*_Stage__3__–vs–Stage__4_ = 0.794 (Figure 3C right). Again, CBX4 tended to be associated with colon cancer early development instead of its late progression. Together, all these data suggested a role of CBX4 as a predictor for poor prognosis.

Given the potential interaction of CBX4 with the circadian clock core genes, we further explored the correlation between the patient survival and the clock gene expression in COAD. While higher level of CLOCK was associated with improved overall survival (Cox *p* < 0.05), increased expression of PER1 (Cox *p* < 0.01), PER3 (Cox *p* < 0.05), and CRY2 (Cox *p* < 0.05) predicted poor prognosis (Figure 3A lower). These results were consistent with their coordinated expression with CBX4.

### CBX4 Impacts Immunophenotype in Colon Cancer

Tumor infiltrating lymphocytes (TILs) are recognized to predict lymphatic metastasis and survival in cancers (Azimi et al., 2012). We next investigated how immune infiltration levels were affected by CBX4 expression. We assessed the correlations of CBX4 expression with tumor immune infiltration abundance in the 40 cancer types from the Gene module of TIMER2.0. Among the diverse infiltrates, we found that CBX4 expression was shown to have significant correlations with infiltrating B cells in 33 types (Figure 4A), infiltrating CD4^+^ T cells in 36 types (Figure 4B), myeloid derived suppressor cells (MDSCs) in 17 types (Figure 4C) and cancer associated fibroblast (CAFs) in 28 types of the 40 cancers (Figure 4D) (*p* < 0.05). Given the multiple potential correlations of CBX4 expression with the infiltration levels in diverse types of tumors, we next focused on colon adenocarcinoma for further investigations. As shown in Figure 4E, CBX4 was negatively correlated with infiltrating B cells (Spearman’s rho = −0.134, *p* < 0.05) and CD4 + T cells (Spearman’s rho = −0.176, *p* < 0.01) including CD4 + Th2 cells (Spearman’s rho = −0.213, *p* < 0.001). Conversely, significant positive correlations were observed with MDSC (Spearman’s rho = 0.109, *p* < 0.05) and CAFs (Spearman’s rho = 0.183, *p* < 0.001). In view of the potential interactions between CBX4 and these immune infiltrates in COAD, we further evaluated the association of these tumor immune subsets with the clinical outcome via the Survival module of TIMER. The Kaplan-Meier plots were output to visualize the survival differences between high and low levels of infiltrating B cells, CD4 + Th2 cells, MDSC and CAFs. Infiltrating B cells (*p* < 0.05) and CD4 + Th2 cells (*p* < 0.05) were both significantly associated with increased survival rate, while MDSC (*p* < 0.05) and CAFs (*p* < 0.05) predicted poor prognosis in COAD (Figure 4G). Furthermore, the COAD patients were dichotomized based on the CBX4 mRNA expression levels (FPKM) of the TCGA cases listed in Supplementary Table 1. The corresponding immune scores were profiled on the RNA-seq-V2 platform of the ESTIMATE algorithm. We found that CBX4 expression levels were associated with relatively low immune scores (Figure 4F).

In addition to the major cellular composition of the immune infiltrates, we investigated the relationship of CBX4 expression and a compendium of gene representatives of various CD4^+^ T cell subsets, including Th1, Th2, Th17, and regulatory T cells, as proposed by Charoentong and colleagues (Charoentong et al., 2017). As illustrated in Tables 1, 2, CBX4 was partially associated with some gene markers of Th1 and Th17 cells, whereas it is worth noting that its expression level was strong- positively correlated with Th2 – associated transcriptional factor GATA-3 (*p* < 0.001) and STAT6 (*p* < 0.001) which function to stably commit differentiating cells toward the Th2 phenotype. In addition, CBX4 showed significant positive correlations with those T cell exhaustion and Treg-associated parameters, such as PD-1 (*p* < 0.01), TGFβ (*p* < 0.001), Foxp3 (*p* < 0.05), L1CAM (*p* < 0.001) and PTGIR (*p* < 0.001), as well as the markers of MDSC, such as CD11b (*p* < 0.01) (Charoentong et al., 2017) (Table 1 and Supplementary Figure 2). These interactions were further confirmed by using the GEPIA database (Table 2 and Supplementary Figure 3). Consistent with their coordinated expression with CBX4, PER1, and PER3, the key members of the PER/CRY-controlled negative loop of the circadian clock, showed strong positive correlations with these gene representatives in COAD (Table 3). These data suggested a potential role of CBX4 and deregulated circadian rhythm in immune escape in the colon cancer microenvironment.

## Discussion

Here we demonstrate that CBX4 may contribute to colon cancer development via the potential interactions with the circadian rhythm genes. As illustrated in Figure 2, CBX4 expression is negatively correlated with that of CLOCK. At the first glance, this result may be interpreted as the transcriptional repression of CLOCK imposed by CBX4, a key component of the PRC1 repressor complex. In such a scenario, one would expect the accompanied down-regulation of downstream clock controlled genes, which is contradictory to the observation of positive correlation of PER1, PER3, CRY2, and NR1D1. As such, we speculate that there is another possibility that the interactions are more likely dependent on the SUMO E3 ligase activity of CBX4. Previous studies have reported that the two SUMO-interacting motifs (SIMs) of CBX4 protein contribute to its SUMO E3 ligase-dependent functions (Luis et al., 2011). SUMO E3 ligases mainly act as adapters to recruit the E2 to the substrates (Kagey et al., 2003). Here, whether CBX4 may directly or indirectly function to bridge E2 and SUMO isoforms to the CLOCK/ARNTL substrates and initiate dynamic post-translational regulation has become our concern.

Heterodimers of CLOCK/ARNTL drive rhythmic expression of clock-controlled genes, thereby mediating circadian physiological behaviors. Colon function is controlled and optimized by endogenous circadian clock, allowing the anticipation of the chyme appearance at certain time (Polidarova et al., 2017). Interventions to the key regulators of the circadian rhythm feedback loops are associated with disorders in colon function, leading to increased susceptibility for developing colon cancer. Numerous studies have emphasized the role of post-translational modifications of the circadian rhythm components in regulating the molecular circadian machinery (Lee et al., 2008). CLOCK has been identified to be a substrate of SUMO, and the sumoylation sites of CLOCK are the highly conserved lysine residues K67 and K851 which are located in the bHLH/PAS and C-terminal regions, respectively (Li et al., 2013). K67 is essential for the associations of CLOCK with other proteins, such as ARNTL, PER1, and CRY1. K851 also exerts regulatory effects on CLOCK activity (Li et al., 2013). These SUMO modifications are found to potentiate the transcriptional activity of the CLOCK/ARNTL complex by stimulating its E-box binding activity (Lee et al., 2008; Li et al., 2013). Thus this might suggest such an interpretation of our observations that up-regulated CBX4 in COAD might directly or indirectly enhance the transcriptional activity of the CLOCK/ARNTL complex through SUMO modification, which in turn activates the downstream clock controlled genes. Accumulation of PERs and CRYs exerts a feedback inhibition to the CLOCK/ARNTL complex. Therefore, the level of CLOCK might be determined by the enhanced inhibitory feedback effect of the downstream clock controlled genes, rather than direct transcriptional suppression induced by CBX4. Although further delineation is needed to support such a mechanism, this scenario will provide an alternate functional avenue of CBX4.

CBX4 was associated with a poor prognosis in COAD, whereas the circadian clock genes correlated with differential clinical outcomes as illustrated in Figure 3. CLOCK, negatively correlated with CBX4, was associated with good overall survival. On the other hand, PER1, PER3, CRY2 which were positively correlated with CBX4, were associated with poor prognosis. Circadian clock homeostasis is believed to have a tumor protective role (Savvidis and Koutsilieris, 2012; Labrecque and Cermakian, 2015). When each circadian clock core gene is subject to aberrant regulation, they might perform differential actions in certain context of tumor microenvironment. Interventions to the key regulators of molecular circadian clock may contribute to disorders in circadian homeostasis, which eventually lead to changes in cellular functions.

As an immunogenic tumor, colon adenocarcinoma is characterized by a strong intrinsic immune suppressive microenvironment as well as a high immune evasion, which may represent a major impediment for effective immune responses against tumor (Zhang et al., 2020). Therefore, we next unmask a landscape of infiltrating lymphocytes in COAD engaged by CBX4. As illustrated in Figure 4, levels of CBX4 were significantly negatively correlated with infiltrating B cells and CD4^+^ T cells, while positively correlated with MDSCs and CAFs. The role of TILs is highly content and stage dependent. In colon cancer, B cells constitute a significant proportion of the tumor immune infiltrates, characterized by accumulation of terminally memory B cells or plasma cells suggestive of a specific anti-tumor immune response (Shimabukuro-Vornhagen et al., 2014). The abundance of infiltrating B appears to have a beneficial impact on the patient’s clinical outcome as illustrated in Figure 4G. CD4 + Th cells target tumor cells either directly through cytolytic mechanisms or indirectly by modulating the tumor microenvironment (TME) (Borst et al., 2018). CD4 + Th1 and Th17 cells have been well documented to activate cytotoxic T lymphocytes, whereas Tregs inhibit efficacious anti-tumor responses (Sun et al., 2002). Here, CD4 + Th2 infiltration also appeared to have a favorable prognosis in COAD when the follow-up time was started from 13months (Figure 4G), and the infiltration level was negatively correlated with CBX4 (Figure 4E). As inhibitory regulatory cells, MDSCs attenuate antitumor immunity by suppressing T cell activation and perturbing innate immune cells (Zhang et al., 2020). By secreting growth factors, cytokines, and chemokines, CAFs in TME facilitate cancer cell proliferation, metastasis, angiogenesis and drug resistance, and shield tumor cells from immune surveillance by enhancing key immune checkpoints (Zhang et al., 2020). The significant positive correlations of CBX4 with these two immune subsets suggested a possible role of CBX4 in immune escape in the colon cancer microenvironment.

The implication of CBX4 in the regulation of tumor immunity was further supported by analyzing the correlations between CBX4 expression and the gene representatives of various functional T cell and MDSC subsets in COAD. Strikingly, we noticed that CBX4 showed strong positive correlations with a variety of parameters characteristic of T cell exhaustion, regulatory T cells and MDSCs (Tables 1, 2), which supported a potential involvement of CBX4 in immuno-suppression. Furthermore, significant positive correlations of CBX4 with STAT1 and IL-6R/STAT3 were observed, and the activation of STAT1 and IL-6/STAT3 signaling has been reported to promote immunosuppression in the TME of colon cancer (Zhang et al., 2020). However, pivotal Th1 regulatory cytokine IFN-γ and another key Th17 -associated transcriptional factor RORγt were not affected by CBX4. In addition, we note that CBX4 was strongly correlated with Th2 – associated transcriptional factor GATA-3 and STAT6 which function to stably commit differentiating cells toward the Th2 phenotype. Therefore, CBX4-expressing tumors appear to be featured by an immune response biased toward type 2. Again, it is interesting to note that PER1 and PER3, consistent with their coordinated expression with CBX4, showed strong positive correlations with these immune metagenes as well, suggesting a possible interaction between CBX4 and the circadian machinery.

## Conclusion

Despite extensive studies on potential mechanisms of CBX4-mediated tumorigenesis, CBX4’s functional panorama remains to be explored. Circadian rhythm disruption is associated with higher cancer risk, whereas delineation of the mechanisms linking circadian rhythm disruption to cancers has yet to be elaborated. Little is known about the relationship between CBX4 and circadian rhythm genes in colon cancer as well as the potential impacts on the tumor immunity. Despite still a preliminary stage of this study, our data suggest that CBX4 is up-regulated and associated with poor clinical outcome in COAD. It is significantly correlated with the circadian clock core genes, and impacts tumor immune infiltration, supporting a potential involvement in immune escape in the colon tumor microenvironment. We anticipate our findings will provide a framework for future studies that will further elucidate in-depth molecular mechanism of CBX4-mediated circadian disruption, which may provide better insights for development of new targeting strategies.

## Data Availability Statement

The datasets presented in this study are available in online repositories. These data were derived from the following publicly available resources:

1. TIMER: https://cistrome.shinyapps.io/timer/,
2. UALCAN: Ualcan.path.uab.edu/analysis
3. STRING: https://string-db.org/cgi/input?sessionId=bnvX D8cMolD6&input_page_active_form=multiple_identifiers
4. cBioportal: https://www.cbioportal.org/
5. GEPIA: http://gepia.cancer-pku.cn
6. PrognoScan: http://dna00.bio.kyutech.ac.jp/PrognoScan/
7. ESTIMATE: http:// bioinformatics.mdanderson.org/estimate/.

## Author Contributions

WW designed the experiments, collected the data, and wrote the manuscript. WZ contributed to data organization and technical assistance. YZ supervised the project, edited the manuscript, and provided overall guidance. All authors approved the submitted version.

## Conflict of Interest

The authors declare that the research was conducted in the absence of any commercial or financial relationships that could be construed as a potential conflict of interest.

## Footnotes

1 http://ualcan.path.uab.edu

2 http://cbioportal.org

## Supplementary Material

Supplementary Figure 1 The Genomic Alterations of CBX4 across Cancers. Proportions of CBX4 mutations and somatic copy number alterations including amplification and deletion in TCGA samples were analyzed via cBioportal, based on TCGA Pan Cancer Atlas studies in which 594 of colorectal carcinoma cases were included. The Green bars indicate non-synonymous mutations, red bars are gene amplifications, blue bars are deep deletions, and gray bars indicate multiple alterations.

Supplementary Figure 2 Correlations between CBX4 and immune marker sets of various functional immune cells in COAD determined by TIMER2.0. The purity-adjusted partial spearman’s rho value represents the degree of the correlation, and the significance was indicated.

Supplementary Figure 3 Correlations between CBX4 and gene representatives of various functional cell subsets in COAD determined by GEPIA. COAD tumor: colon adenocarcinoma tissue in TCGA; COAD normal: adjacent normal tissues in TCGA. Spearman’s and Pearson’s R and the significance were indicated.
