# Supplementary material for: CBX4 Provides an Alternate Mode of Colon Cancer Development via Potential Influences on Circadian Rhythm and Immune Infiltration
Source: Front Cell Dev Biol. 2021 Jun 7;9:669254. doi: 10.3389/fcell.2021.669254 (PMC8253160; doi:10.3389/fcell.2021.669254)
Supplement: Supplementary Figure 1 — The Genomic Alterations of CBX4 across Cancers. Proportions of CBX4 mutations and somatic copy number alterations including amplification and deletion in TCGA samples were analyzed via cBioportal, based on TCGA Pan Cancer Atlas studies in which 594 of colorectal carcinoma cases were included. The Green bars indicate non-synonymous mutations, red bars are gene amplifications, blue bars are deep deletions, and gray bars indicate multiple alterations. [file Data_Sheet_1.pdf]

Supplementary Figure. 1

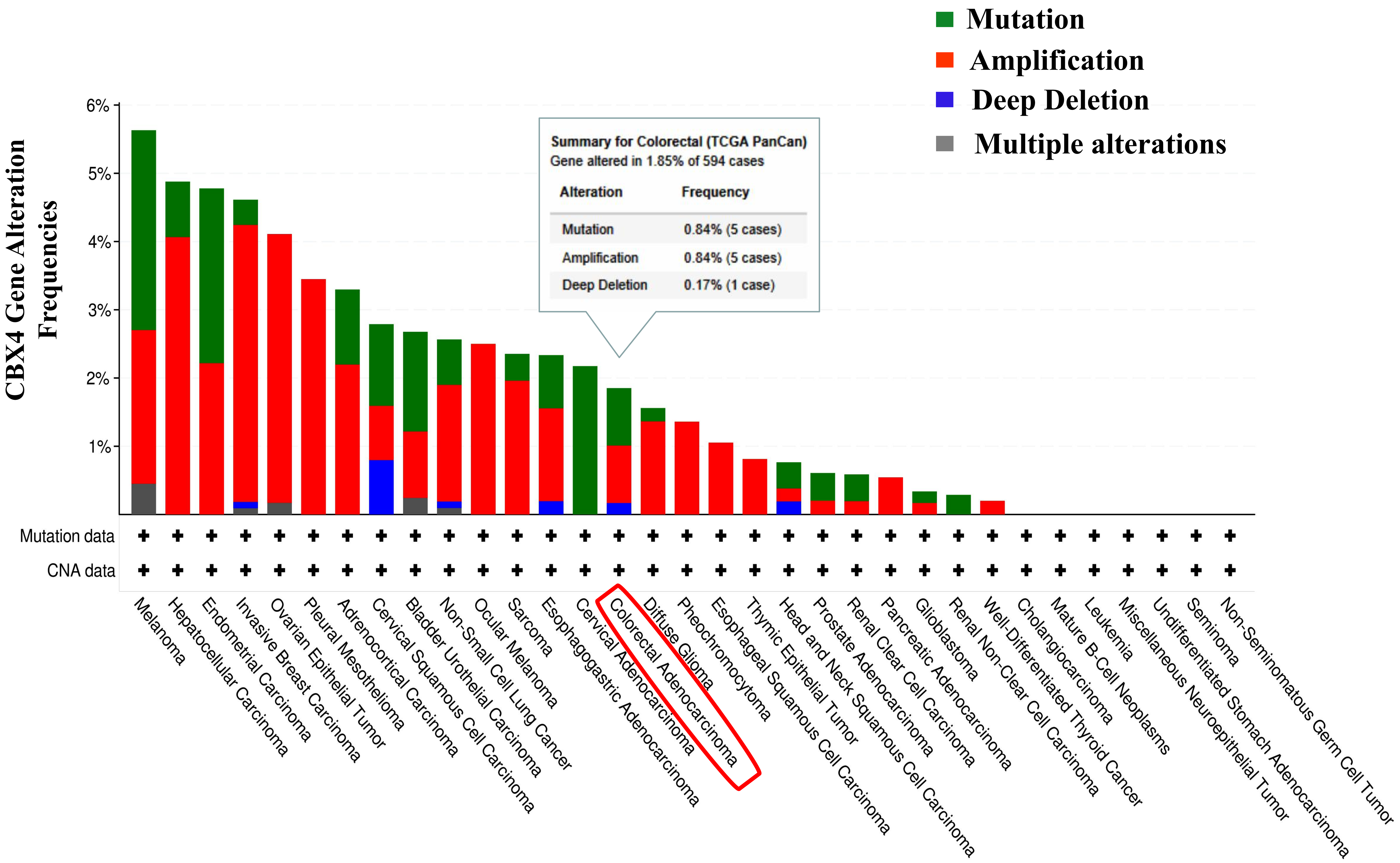

Supplementary Figure. 2

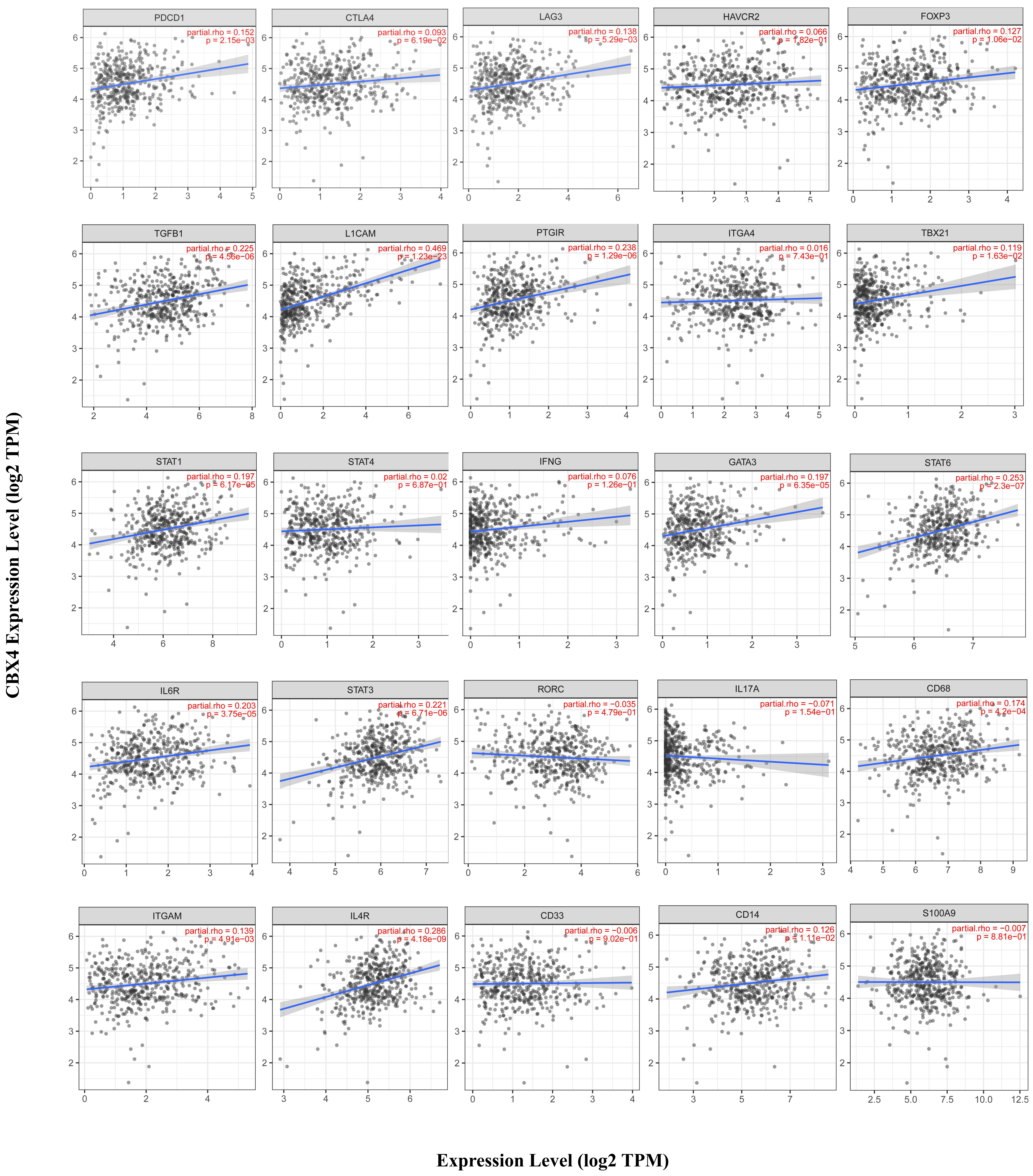

Supplementary Figure. 3

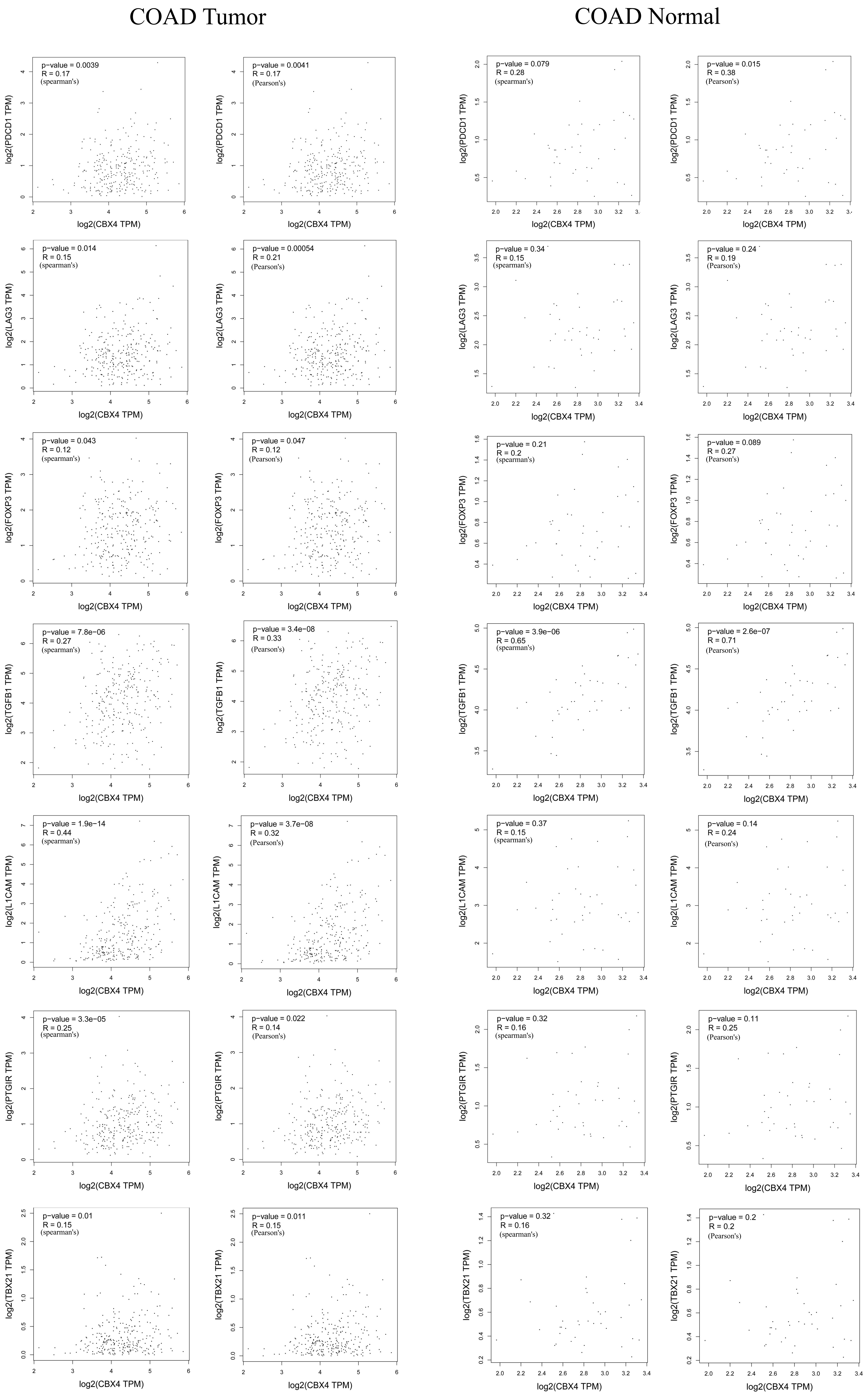

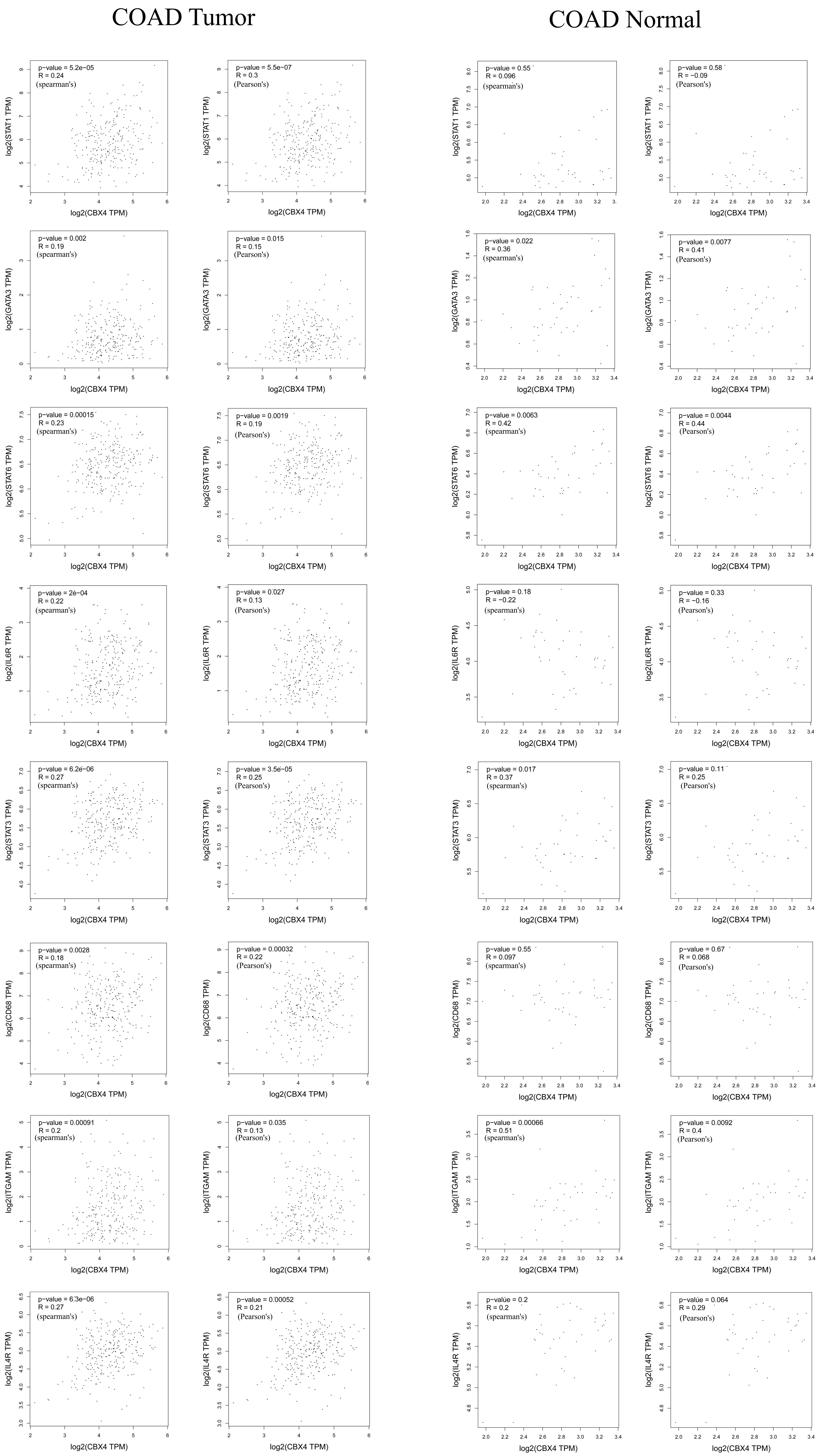
COAD Tumor
COAD Normal
log2(STAT1 TPM)
log2(GATA3 TPM)
log2(STAT6 TPM)
log2(IL6R TPM)
log2(STAT3 TPM)
log2(CD68 TPM)
log2(ITGAM TPM)
log2(IL4R TPM)
log2(CBX4 TPM)
p−value = 5.2e−05 R = 0.24 (spearman's)
p−value = 5.5e−07 R = 0.3 (Pearson's)
p−value = 0.55 R = 0.096 (spearman's)
p−value = 0.58 R = −0.09 (Pearson's)
p−value = 0.002 R = 0.19 (spearman's)
p−value = 0.015 R = 0.15 (Pearson's)
p−value = 0.022 R = 0.36 (spearman's)
p−value = 0.0077 R = 0.41 (Pearson's)
p−value = 0.00015 R = 0.23 (spearman's)
p−value = 0.0019 R = 0.19 (Pearson's)
p−value = 0.0063 R = 0.42 (spearman's)
p−value = 0.0044 R = 0.44 (Pearson's)
p−value = 2e−04 R = 0.22 (spearman's)
p−value = 0.027 R = 0.13 (Pearson's)
p−value = 0.18 R = −0.22 (spearman's)
p−value = 0.33 R = −0.16 (Pearson's)
p−value = 6.2e−06 R = 0.27 (spearman's)
p−value = 3.5e−05 R = 0.25 (Pearson's)
p−value = 0.017 R = 0.37 (spearman's)
p−value = 0.11 R = 0.25 (Pearson's)
p−value = 0.0028 R = 0.18 (spearman's)
p−value = 0.00032 R = 0.22 (Pearson's)
p−value = 0.55 R = 0.097 (spearman's)
p−value = 0.67 R = 0.068 (Pearson's)
p−value = 0.00091 R = 0.2 (spearman's)
p−value = 0.035 R = 0.13 (Pearson's)
p−value = 0.00066 R = 0.51 (spearman's)
p−value = 0.0092 R = 0.4 (Pearson's)
p−value = 6.3e−06 R = 0.27 (spearman's)
p−value = 0.00052 R = 0.21 (Pearson's)
p−value = 0.2 R = 0.2 (spearman's)
p−value = 0.064 R = 0.29 (Pearson's)

Suppl. Table. 1. TCGA colon adenocarcinoma cases

Case #	Case ID	Case #	Case ID	Case #	Case ID	Case #	Case ID	Case #	Case ID
1	TCGA-AA-A004-01	46	TCGA-AG-3587-01	91	TCGA-AA-3941-01	136	TCGA-AG-3887-01	181	TCGA-AA-A02O-01
2	TCGA-AG-4007-01	47	TCGA-AG-A025-01	92	TCGA-AA-3968-01	137	TCGA-AA-3977-01	182	TCGA-CK-4951-01
3	TCGA-AG-A02G-01	48	TCGA-AA-3848-01	93	TCGA-CM-4747-01	138	TCGA-AA-3994-01	183	TCGA-AA-3502-01
4	TCGA-AG-A016-01	49	TCGA-AA-3867-01	94	TCGA-CM-4752-01	139	TCGA-AA-3855-01	184	TCGA-A6-2684-01
5	TCGA-AG-A026-01	50	TCGA-AA-A01X-01	95	TCGA-AA-3488-01	140	TCGA-AA-3664-01	185	TCGA-AG-3575-01
6	TCGA-AG-A002-01	51	TCGA-AA-A01T-01	96	TCGA-A6-3810-01	141	TCGA-AG-3599-01	186	TCGA-AA-3514-01
7	TCGA-A6-2671-01	52	TCGA-AA-A029-01	97	TCGA-AA-A01V-01	142	TCGA-A6-4107-01	187	TCGA-AA-3989-01
8	TCGA-AA-A00L-01	53	TCGA-AA-3517-01	98	TCGA-AA-A01S-01	143	TCGA-AF-2692-01	188	TCGA-CM-4746-01
9	TCGA-CM-4748-01	54	TCGA-A6-2680-01	99	TCGA-AA-3494-01	144	TCGA-AG-3598-01	189	TCGA-AG-3611-01
10	TCGA-AG-3885-01	55	TCGA-AA-3980-01	100	TCGA-AG-4008-01	145	TCGA-AA-A00Q-01	190	TCGA-AA-3851-01
11	TCGA-AG-3727-01	56	TCGA-AA-A010-01	101	TCGA-AA-3549-01	146	TCGA-AA-3562-01	191	TCGA-AA-3520-01
12	TCGA-AA-A00E-01	57	TCGA-AA-A00U-01	102	TCGA-AA-3976-01	147	TCGA-AA-3864-01	192	TCGA-AA-3673-01
13	TCGA-A6-2679-01	58	TCGA-AG-4005-01	103	TCGA-AG-A015-01	148	TCGA-AG-3896-01	193	TCGA-AG-3586-01
14	TCGA-AG-3726-01	59	TCGA-AA-3519-01	104	TCGA-AA-A03F-01	149	TCGA-AA-3524-01	194	TCGA-AA-3866-01
15	TCGA-A6-3807-01	60	TCGA-AA-3811-01	105	TCGA-AA-A01F-01	150	TCGA-AA-3666-01	195	TCGA-AZ-4315-01
16	TCGA-AG-A014-01	61	TCGA-AF-2691-01	106	TCGA-AA-A00A-01	151	TCGA-AA-3952-01	196	TCGA-AA-3819-01
17	TCGA-AG-3909-01	62	TCGA-AA-3518-01	107	TCGA-AZ-4684-01	152	TCGA-AA-3842-01	197	TCGA-AG-3892-01
18	TCGA-A6-2681-01	63	TCGA-AA-3856-01	108	TCGA-AG-4001-01	153	TCGA-AG-3894-01	198	TCGA-AA-3492-01
19	TCGA-AA-3812-01	64	TCGA-A6-2685-01	109	TCGA-AA-A00N-01	154	TCGA-AA-3530-01	199	TCGA-AA-3956-01
20	TCGA-AA-3667-01	65	TCGA-AA-A017-01	110	TCGA-AG-A00Y-01	155	TCGA-AA-3710-01	200	TCGA-AA-3831-01
21	TCGA-AA-A01G-01	66	TCGA-AA-3552-01	111	TCGA-AA-3548-01	156	TCGA-A6-2674-01	201	TCGA-AA-3982-01
22	TCGA-AA-A02H-01	67	TCGA-AG-3898-01	112	TCGA-AA-3692-01	157	TCGA-AA-3854-01	202	TCGA-AA-3869-01
23	TCGA-AA-3506-01	68	TCGA-AA-3531-01	113	TCGA-A6-2676-01	158	TCGA-AG-3584-01	203	TCGA-A6-2678-01
24	TCGA-AG-3999-01	69	TCGA-AG-3608-01	114	TCGA-AA-3858-01	159	TCGA-AA-A00K-01	204	TCGA-AA-A01I-01
25	TCGA-AA-3870-01	70	TCGA-AA-3553-01	115	TCGA-AA-A01D-01	160	TCGA-AA-3875-01	205	TCGA-AA-3509-01
26	TCGA-AZ-4614-01	71	TCGA-AA-A02W-01	116	TCGA-AA-3542-01	161	TCGA-AA-3532-01	206	TCGA-AA-3495-01
27	TCGA-AA-3846-01	72	TCGA-AG-A00C-01	117	TCGA-AG-3883-01	162	TCGA-AF-3400-01	207	TCGA-CA-5256-01
28	TCGA-AZ-4615-01	73	TCGA-AG-3578-01	118	TCGA-AA-3510-01	163	TCGA-AA-3522-01	208	TCGA-AA-3947-01
29	TCGA-AA-A02J-01	74	TCGA-AA-3678-01	119	TCGA-AA-3679-01	164	TCGA-AA-3681-01	209	TCGA-CM-5341-01
30	TCGA-AA-3975-01	75	TCGA-AA-A01C-01	120	TCGA-A6-3809-01	165	TCGA-AG-3593-01	210	TCGA-AA-3560-01
31	TCGA-AG-A011-01	76	TCGA-AA-A01Q-01	121	TCGA-AA-3877-01	166	TCGA-AA-3986-01		
32	TCGA-AA-A00J-01	77	TCGA-AG-3600-01	122	TCGA-AA-3815-01	167	TCGA-AG-3901-01		
33	TCGA-AG-3612-01	78	TCGA-AA-3555-01	123	TCGA-AA-3930-01	168	TCGA-AA-3561-01		
34	TCGA-AG-3890-01	79	TCGA-AY-4071-01	124	TCGA-AA-3950-01	169	TCGA-AA-3939-01		
35	TCGA-AA-A03J-01	80	TCGA-AA-3696-01	125	TCGA-AG-A036-01	170	TCGA-AA-3516-01		
36	TCGA-AA-3833-01	81	TCGA-A6-2683-01	126	TCGA-AG-3893-01	171	TCGA-AA-3818-01		
37	TCGA-AG-A02X-01	82	TCGA-AA-3538-01	127	TCGA-AG-3574-01	172	TCGA-AG-A02N-01		
38	TCGA-CM-4750-01	83	TCGA-AA-A01K-01	128	TCGA-AG-3581-01	173	TCGA-AA-3688-01		
39	TCGA-AA-A00W-01	84	TCGA-AA-3680-01	129	TCGA-AA-3862-01	174	TCGA-AA-3861-01		
40	TCGA-AA-A00Z-01	85	TCGA-AA-3821-01	130	TCGA-A6-3808-01	175	TCGA-AA-3556-01		
41	TCGA-AY-4070-01	86	TCGA-AG-A023-01	131	TCGA-AA-3837-01	176	TCGA-A6-2672-01		
42	TCGA-AA-3841-01	87	TCGA-A6-2682-01	132	TCGA-AG-3580-01	177	TCGA-AA-3970-01		
43	TCGA-AA-3979-01	88	TCGA-AG-3582-01	133	TCGA-AG-3609-01	178	TCGA-AA-3971-01		
44	TCGA-AA-3525-01	89	TCGA-AA-3973-01	134	TCGA-AZ-4308-01	179	TCGA-AG-3605-01		
45	TCGA-AA-A00O-01	90	TCGA-AA-3860-01	135	TCGA-AG-3583-01	180	TCGA-AA-3844-01		
